# The Contentious History of Sirtuin Debates

**DOI:** 10.5041/RMMJ.10093

**Published:** 2012-10-31

**Authors:** Shoshana Naiman, Haim Y. Cohen

**Affiliations:** The Mina & Everard Goodman Faculty of Life Sciences, Bar-Ilan University, Ramat-Gan 52900, Israel

**Keywords:** Aging, deacetylation, dietary restriction, Sir2, SIRT6, sirtuins

## Abstract

The sirtuins are highly conserved enzyme homologues of the yeast Sir2, with activities of NAD+ dependent deacetylase and/or mono ADP ribosyltransferase. A long line of evidence has implicated sirtuins in regulating the aging process of yeast, worms, flies, and rodents. Moreover, much work has been published on the important role of sirtuins in several age-related diseases such as diabetes type II, cancer, cardiovascular diseases, and dyslipidemia. However, despite the many publications supporting a pro-longevity role for sirtuins, there has been emerging debate about the direct role of *Caenorhabditis elegans* and *Drosophila melanogaster* sirtuins in aging and in lifespan extension in response to dietary restriction. In addition, until recently, the role of the seven mammalian sirtuins, SIRT1 to SIRT7, in regulating lifespan was unclear. Here, we review the history of the scientific debate on the role of sirtuins in regulating lifespan, especially in light of a recent publication showing a direct regulation of mammalian lifespan by a sirtuin family member, SIRT6.

From the beginning of humanity, we have been seeking an explanation and consolation for the continuous physiological decline of aging. These days were well described in Ecclesiastes 12:1 as “… and years arrive, about which you will say, I have no desire in them.” The significant increase in human longevity during the last century has created great sociological, economic, and mainly medical challenges. To answer these challenges, one must understand and control the mechanisms that determine the rate of aging. In essence, most accepted theories on the mechanism of aging, such as the “snowball,” “free radicals,” and “disposable soma,” share a common denominator in suggesting that improved body maintenance could extend life. Particularly important are increasing genome stability and preserving proper metabolism.

One family of proteins that have been implicated in aging and the regulation of metabolism and genome stability are the sirtuins. The sirtuins are highly conserved enzyme homologues of the yeast Sir2 protein,^1^ with activities of NAD+ dependent deacetylase and/or mono ADP ribosyltransferase^2^–^4^ that were found to be pivotal in the regulation of longevity. Strikingly, although sirtuins have been studied for over a decade, the scientific field is still arguing about the role of sirtuins in regulating longevity. This long-time debate is summarized herein, together with an explanation regarding the current knowledge of this issue.

The discovery of sirtuins as regulators of aging began in yeast. Several studies originally reported that a yeast protein, namely silence information regulator 4 (Sir4), is involved in the regulation of yeast lifespan. Yeast carrying a mutation in Sir4 has extended lifespan along with short telomeres.^5^ These observations led to the conclusion that in the absence of normal telomere length, Sir4 localizes to an unknown aging regulator locus. Later on, this site was recognized in the yeast genome as the rDNA locus, a tandem repeat of the coding sequences for the ribosomal RNA (rRNA).^6^ This knowledge led to the discovery that the basis for yeast aging is the recombination events within rDNA that release a single repeat in its circular form, since the extrachromosomal rDNA circle (ERC) can exponentially accumulate and kill the cell.^7^ Soon after, it was shown that Sir2, a member of the Sir4 complex, regulates the rate of ERC creation and therefore the rate of yeast aging.^8^

In the late 1990s a study from the Guarente lab, led mainly by Matt Kaeberlein, demonstrated that deletion of Sir2 shortens yeast lifespan and that Sir2 overexpression extends yeast lifespan.^8^ However, a possible explanation of the mechanism by which Sir2 regulates yeast aging came after an elegant study by Shin Imai and Lenny Guarente that revealed for the first time the true enzymatic activity of Sir2—a NAD+ dependent histone deacetylase.^9^ Moreover, another study showed that deletion of Sir2 blocked the beneficial effects of dietary restriction (DR) on lifespan.^10^ The latter observation suggests that sirtuins were required for the DR-mediated increase in lifespan.

Dietary restrictions or reducing caloric intake by 30% were shown to extend the lifespan of many organisms from yeast to rodents. Moreover, the lifespan extension was accompanied with increased health-span, expressed by decreased incidence of tumorigenesis, diabetes type II, and other age-related diseases.^11^

However, whether DR also affects primates is currently under debate, as two recent studies on rhesus monkeys fed a DR diet published contradicting results regarding DR-mediated increase in lifespan.^12^,^13^ While one study showed that DR significantly increased lifespan, the other failed to find an effect. These results may be due to dietary differences between the studies, or the origin of the monkeys. Thus, even before sirtuins entered the picture, possible treatments to extend lifespan were fraught with debate and conflict.

Thus, given the evolutionary conservation of the positive effects of DR on lifespan from yeast to rodents, and the possible effect in monkeys, it is important to explore how DR is regulated in order to translate this knowledge into human therapy. For example, an impressive study carried out by Nir Barzilai’s group showed that removal of white adipose tissues (WAT) from rats can mimic the positive effects of DR, suggesting that WAT might mediate the DR response, or that DR regulates WAT.^14^ However, although DR-increased lifespan was discovered over 80 years ago, the detailed mechanisms underlying DR effects are still elusive.

Sir2 and its homologue sirtuins became more attractive to the global aging community when a series of publications demonstrated that sirtuins are pivotal in the regulation of longevity in lower metazoans. Similar to the findings in the yeast *Saccharomyces cerevisiae*, increasing the activity of sirtuins from *Caenorhabditis elegans*^15^ and *Drosophila melanogaster*,^16^ using either genetic^17^ or chemical means,^18^ also extends their lifespan by at least 15%. Therefore, it was suggested that the role of sirtuins in regulating lifespan is evolutionarily conserved, and understanding its regulation at the molecular level thus has great therapeutic opportunities.

How are sirtuins regulated? To date we know of multiple layers of sirtuin regulation. Initially, an intense debate took place on this matter. At first, it was suggested that NAD+ levels regulate yeast Sir2 activity.^19^ However, in yeast, exact measurements showed that cellular concentrations of NAD+ are around 4 mM. Thus, given that the Km of Sir2 for NAD+ is around 50 μM, a 10-fold change in NAD+ concentration was required to affect Sir2 activity.^20^ Therefore, the Sinclair group suggested that sirtuin activity is regulated by nicotinamide (NAM), one of the products of its NAD+ dependent deacetylase enzymatic activity.^21^ On the other hand, it was recognized that fluctuations in NAD levels cannot provide a reasonable model for sirtuin regulation, and the Guarente group suggested that NADH, which exists in the micromolar range in the cell, inhibits Sir2 enzymatic activity.^22^ A recent study showed that yeast Sir2 activity can be regulated by both NADH and NAM: With high DR (0.1% glucose), clearance of NAM regulates Sir2 activity, whereas with low DR (0.5% glucose) a reduced NADH/NAD ratio regulates Sir2 activity.^23^

What controls endogenous NAM levels? In yeast, PNC1 enzyme converts NAM, a sirtuin inhibitor, into nicotinic acid (NA), which does not inhibit Sir2. This NA is later used by the NAD salvage pathway to generate NAD+. A sophisticated study by Anderson et al. demonstrates that PNC1 levels are regulated by nutrient availability.^21^ Under DR conditions, PNC1 levels and activity increase, resulting in increased Sir2 activity. This model was expanded for other mild stressed conditions such as increased osmolarity and heat, in which PNC1-dependent clearance of NAM under various stresses increases yeast lifespan via Sir2 up-regulation. However, soon after this mechanism was published, the theory that the Sir2-PNC1/NAM cycle regulates yeast lifespan attracted criticism, despite the support of data from multiple studies. First, the role of Sir2 as a key regulator of yeast lifespan was challenged.^24^ Second, at that time, no ortholog for PNC1 in other organisms was found. Therefore, the answer as to whether this model is unique only for yeast remains elusive.

The first uncertainty about the role Sir2 plays in modulating replicative longevity in yeast via regulation of the rate of ERC formation arose from the observation that the lifespan extension by overexpressing Sir2 was strain-dependent.^24^ In addition, it was noticed that in the BY4742 yeast strain, mutation of fob1, which blocks the formation of ERCs, or Sir2 overexpression, together with DR has a cumulative effect on yeast lifespan.^24^ Put simply, DR extended the lifespan of fob1 or sir2 double mutation. Thus, at least in this yeast strain the effect of DR cannot be Sir2 or ERC-dependent, as an additional increase of lifespan was seen with each treatment. In response to these claims other groups have shown that in the absence of Sir2 another yeast sirtuin, Hst2, takes over and regulates the positive effect of DR on yeast lifespan via ERC formation.^25^

However, this was not the end of the debate. Soon after, two researchers published that in the BY4742 yeast strain, double mutation of sir2 and fob1 along with a mutation in one of the hst isoforms hst1/hst2/hst4 has no significant effect on the yeast lifespan.^26^ Treatment with DR extended the lifespan of these combinations. Notably, the role of Hst3 in this study was complex. Mutations in hst3 only, or triple mutation sir2hst3fob1, have a small but significant effect on yeast lifespan. However, once combined with hst4 mutation, yeast lifespan was significantly reduced. Moreover, DR was not able to extend the lifespan of yeast carrying mutations in fob1 and all yeast sirtuins. Interestingly, the authors did not report whether DR can extend the lifespan in hst3hst4 double mutations. Thus, the role of hst3 in DR response remains elusive. Taken together, despite extensive research, the question whether Sir2 or other sirtuins regulate yeast lifespan during DR via controlling ERCs formation is still under debate.

Recent studies have reported that Sir2 regulates yeast replicative lifespan by additional rDNA-independent mechanisms. During cytokinesis, the majority of proteins damaged due to oxidative stress are maintained in the mother cell. Nystrom and his associates showed that Sir2 is required for this asymmetric inheritance, and absence of Sir2 results in an inheritance of oxidatively damaged proteins and reduced capacity to respond to oxidative stress in daughter cells.^27^ Others have shown that an age-associated decrease in Sir2 protein levels is accompanied by an increase in histone H4 K16 acetylation and regions.^28^ These age-related Sir2-dependent effects result in compromised transcriptional silencing at the subtelomeric loci and suggest that Sir2 regulates yeast replicative lifespan through the maintenance of telomeric chromatin. More recent studies even link Sir2 function to a well-known conserved regulator of longevity, the TOR (target of rapamycin) pathway, a sensor of nutrient availability.^29^ However, whether TOR signaling modulates these non-rDNA functions of Sir2 is not yet known.^30^

To summarize, even though the role of Sir2 and sirtuins in yeast longevity has been investigated for 15 years, we still lack a deeper understanding of the mechanisms by which Sir2 activity regulates longevity.

In parallel to the extensive research on yeast sirtuins, a whole new field of sirtuin biology in multicellular organisms has emerged. Researchers began investigating the role of Sir2 in worms and flies. While it is generally accepted that Sir2 extends lifespan in yeast, in worms and flies this topic has been hotly debated, with conflicting studies recently published.

The first Sir2 metazoan homologue shown to extend lifespan was in *C. elegans*, where it was found that overexpression causes a 15%–50% increase in lifespan in two separate transgenic lines.^15^ However, later studies by the same researchers found the increase to be smaller.^31^ The reason for this discrepancy was found to be an unlinked mutation in the original worm strain which augmented the lifespan increase unrelated to the SIR2 (wild type gene) transgene. It would now seem that the increase in *C. elegans* is not 50%, but may be a more modest yet still significant 10%–14%. Yet even this more minor lifespan increase is under debate.

After Sir2 was shown to extend lifespan in *C. elegans*, Sir2 was found to extend lifespan in the fly *D. melanogaster*, in all lines examined, by 18% and 29% in males and females, respectively.^17^ These results were later confirmed by a separate research group.^32^

However, the results of the longevity studies in flies and worms have been recently called into question.^33^ Burnett and colleagues performed an investigation into the longevity data of flies and worms, on the basis that many aging studies are not carried out with the appropriate controls. Importantly, they showed that differences in genetic background are critical, and transgene insertion sites must be examined for linkage with any neighboring genes which may have mutagenic effects. Indeed, it was this study which brought to light that the original 50% lifespan increase observed in worms was potentially due to a different mutation unrelated to Sir2. In addition, they initially found an increase in Guarente’s second line of low-copy Sir2 overexpression. Yet, when Burnett and colleagues outcrossed this second line of low-copy Sir2 overexpression six times in order to remove any effects from random transgene insertion, the longevity effect was once again abrogated, suggesting that the overexpression in the second line was also due to unrelated linkage of neighboring genes. When Guarente’s group repeated their experiments in a worm strain that was backcrossed at least six times they observed a 10%–14% lifespan increase. Therefore, the question as to whether Sir2 increases lifespan in *C. elegans* remains unanswered, and further research must be done to elucidate the differences between these studies.

Similar to the experiments repeated in worms, when Burnett and colleagues repeated the experiments in flies using the more appropriate transgenic control (tubulin/GAL4+) in both weak and strong expression of the transgene, they did not find an increase in lifespan. However, they failed to address a previous study which used inducible Sir2 overexpression to extend lifespan. This study used the appropriate controls and still found a lifespan extension, which was dependent on inducible Sir2 levels.^34^ Interestingly, in that paper, increasing dSir2 levels ∼5-fold as compared to ∼3-fold further extended fly lifespan, indicating that different levels of dSir2 overexpression can differentially increase lifespan. Hence, with conflicting data regarding dSir2-regulated lifespan extension in drosophila, one must wonder exactly which differences are responsible for the apparent discrepancies. Regardless, these studies emphasize the need for appropriate controls in lifespan experiments, as well as outcrossing to overcome effects of transgene integration.

Due to the general confusion regarding the role of sirtuins in worms and flies, the question of whether sirtuins regulate lifespan in mammals was more critical than ever. While there is now a great deal of data on yeast, worms, and flies, which are good research models, they are not similar to mammals and humans. Hence, researchers turned towards mice to investigate whether sirtuins can regulate lifespan in mammals, thereby completely bypassing the sirtuin debate in lower metazoans. The original results in mammals were also puzzling. The first sirtuin examined to regulate lifespan in mammals was the most well-known mammalian sirtuin, SIRT1, the closest mammalian homologue to Sir2. However, although moderate overexpression of SIRT1 protected against age-related diseases such as osteoporosis, glucose intolerance, and metabolic syndrome-related cancers, it did not extend lifespan.^35^ Thus, either SIRT1 has no role in regulating lifespan; or under the weak expression of the transgene (threefold) SIRT1 has no effect of longevity, and stronger expression of the transgene may be necessary to achieve lifespan extension.

In parallel to the research carried out with SIRT1, a separate group examined the role of SIRT6 in regulating lifespan. SIRT6 is a nuclear sirtuin known to be involved in DNA repair, inflammation, and metabolism. It seemed a likely candidate for aging, as the absence of SIRT6 in mice caused a severe aging-like phenotype and early death.^36^ However, early death is not necessarily indicative of a role in longevity, as developmental or metabolic defects and not premature aging can cause lethal damage. Therefore, the recent results of an SIRT6 aging study are noteworthy; for the first time it was shown that SIRT6 extends the lifespan of male mice.^37^ Both median and mean lifespan were significantly increased by ∼15%, a similar increase to that found in Sir2-overexpressing flies and worms, and well within the accepted sirtuin longevity data. A possible explanation for the increased lifespan may be due to a significant decrease in levels and signaling of IGF-1, a well-known regulator of aging. Furthermore, this study was performed in two separate lines of mice, to counteract any site-specific effects of insertion of the SIRT6 transgene. Additionally, to ensure the results were not strain-specific, a mixed CB6 strain was used. Therefore, this study conclusively shows that mammalian sirtuins can extend lifespan in mammals. It is important to note that the lifespan extension was only observed in males and not females, and more research must be carried out to understand the exact mechanism by which this occurs.

These data show for the first time that a mammalian sirtuin can regulate longevity, and finally resolved the long-standing debate as to whether sirtuins regulate lifespan in multicellular organisms (see Figure 1 for a graphic summary of the debate’s history). These exciting results in mammals open the way for new aging studies, as there are other mammalian sirtuins similar to SIRT6 which may also be involved in aging, and combining SIRT6 with a different sirtuin may cause a greater increase in lifespan than SIRT6 alone.

**Figure 1:**
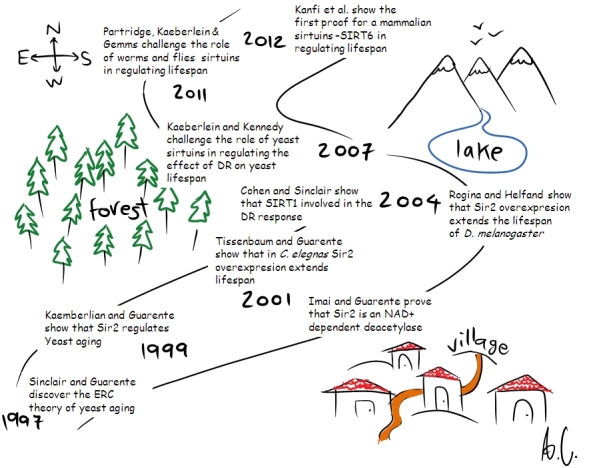
**The history of the debate on the role of sirtuins in regulating lifespan.** DR, dietary restriction; ERC, extrachromosomal rDNA circle; Sir, silent information regulator; SIRT, sirtuin.

In the short period since the discovery of sirtuins, the role they play in regulating lifespan has been highly debated and speculated upon. Results in lower organisms remain uncertain, but the role of mammalian sirtuins in regulating lifespan of mammals has finally been clarified. Although there is still much research to be done to reveal the true nature of sirtuins and their connection to longevity, it is clear that mammalian sirtuins, SIRT6 in particular, are critical to a healthy or long life. Will sirtuins regulate longevity in primates as well? Only time will tell.

## Abbreviations:

DR: dietary restriction;
ERC: extrachromosomal rDNA circle;
NA: nicotinic acid;
NAM: nicotinamide;
rRNA: ribosomal RNA;
Sir: silent information regulator;
SIR2: SIR2 gene;
sir2: mutant SIR2 gene;
Sir2: Sir2 protein;
SIRT: sirtuin;
TOR: target of rapamycin;
WAT: white adipose tissue.
